# LIVER RESECTION IN BRAZIL: A NATIONAL SURVEY

**DOI:** 10.1590/0102-672020180001e1355

**Published:** 2018-06-21

**Authors:** Gilton Marques FONSECA, Vagner Birk JEISMANN, Jaime Arthur Pirola KRUGER, Fabricio Ferreira COELHO, Andre Luis MONTAGNINI, Paulo HERMAN

**Affiliations:** 1 Department of Gastroenterology, Faculty of Medicine, University of São Paulo; 2 Brazilian Chapter of the International Hepato-Pancreato-Biliary Association, São Paulo, SP, Brazil

**Keywords:** Hepatectomy, Liver, Surveys and questionnaires, Laparoscopy., Fígado, Pesquisa, Laparoscopia, Hepatectomia

## Abstract

**Background:**

Liver surgery has developed significantly in the past decades. In Brazil, the interest on it has grown significantly, but there is no study regarding its clinical practice. Despite intrinsic limitations, surveys are well suited to descriptive studies and allow understanding the current scenario.

**Aim:**

To provide an overview on the current spread of liver surgery in Brazil, focusing on groups´ profile, operative techniques and availability of resources.

**Method:**

From May to November 2016, was conducted a national survey about liver surgery profile in Brazil composed by 28 questions concerning surgical team characteristics, technical preferences, surgical volume, results and available institutional resources. The survey was sent by e-mail to 84 liver surgery team leaders from different centers including all regions of the country.

**Results:**

Forty-three study participants (51.2%), from all Brazilian regions, responded the survey. Most centers have residency/fellowship programs (86%), perform and do laparoscopic procedures (91%); however, laparoscopy is still responsible for a little amount of surgeries (1-9% of laparoscopic procedures over all liver resections in 39.5% of groups). Only seven centers (16.3%) perform more than 50 liver resections/year. Postoperative mortality rate is between 1-3% in 55% of the centers.

**Conclusion:**

This is the first depiction of liver surgery in Brazil. It showed a surgical practice aligned with worldwide excellence centers, concentrated on hospitals dedicated to academic practice.

## RESUMO

**Racional:**

A cirurgia hepática se desenvolveu significativamente nas últimas décadas. No Brasil, apesar do crescente interesse pela cirurgia do fígado, não há estudos a respeito da sua prática no país. Assim, foi realizada uma pesquisa do tipo inquérito, apropriada para estudos descritivos, permitindo entender adequadamente o cenário atual nacional.

**Objetivo:**

Obter uma visão abrangente da cirurgia hepática no Brasil, com foco no perfil dos grupos, técnicas operatórias e disponibilidade de recursos.

**Método:**

De abril a novembro de 2016, foi realizado um inquérito nacional sobre o perfil da cirurgia hepática no Brasil, composta por 28 questões relativas às características das equipes cirúrgicas, preferências técnicas, volume cirúrgico, resultados e disponibilidade de recursos nas instituições. A pesquisa foi enviada por e-mail para 84 cirurgiões hepáticos brasileiros líderes de seus respectivos grupos, abrangendo todas as regiões do país.

**Resultados:**

Quarenta e três participantes (51.2%) responderam à pesquisa (42 respostas completas e uma incompleta) de todas as regiões do Brasil. A maioria dos centros tem programa de residência (86%) e faz procedimentos por via laparoscópica (91%); a despeito da laparoscopia corresponder a uma pequena porcentagem das operações (1-9% dos procedimentos são laparoscópicos em 39,5% dos grupos). Apenas sete centros (16.3%) realizam mais do que 50 resecções hepáticas/ano. A taxa de mortalidade pós-operatória é de 1-3% em 55% dos centros.

**Conclusão:**

Esta é a primeira avaliação da cirurgia hepática no Brasil e indica que as práticas cirúrgicas estão alinhadas com centros de excelência mundiais, concentradas em hospitais dedicados à prática acadêmica.

## INTRODUCTION

Liver surgery has developed significantly in the past decades. Comprehension about liver anatomy, development of parenchyma transection techniques, incorporation of new technologies and instruments made liver surgery a complex and effective specialty^5^, requiring structure available in tertiary centers^4^. In the last years, minimally invasive techniques were incorporated to liver surgery, adding a new set of complex operations to be learned and performed by liver surgeons^8^.

In Brazil, the interest in liver surgery has been growing in recent years leading to an increase in performance of hepatic resections throughout the country, although there are challenges to provide access for all patients requiring this kind of procedure^2^ ^,^ ^3^ ^,^ ^12^. Knowledge of the profile of liver surgery in a continental developing country can provide valuable information to guide actions toward improvement in training and acquisition of resources.

Despite intrinsic limitations, surveys provide a “snapshot of how things are at a specific time”^22^, well suited to descriptive studies, allowing to understand current scenario and to search for trends in specific subjects.

Since there is no study about the clinical practice of liver surgery in Brazil, it was conducted a survey including the most relevant groups in hepatic surgery. 

The aim of this study was to provide an overview on the current spread of liver surgery in Brazil, focusing on groups´ profile, operative techniques including minimally invasive procedures and availability of resources.

## METHODS

It was conducted a national survey about liver surgery profile in Brazil, using Redcap electronic data capture tools^8^, from May to November 2016. The survey was sent by e-mail, with a cover letter calling for participation and a hyperlink to the survey, to members of the Brazilian Chapter of the International Hepato-Pancreato-Biliary Association (CB-IHPBA) including all regions of the country. The study was designed to understand the institutional profile, so only one expert surgeon from each center was invited to take part of the study.

The survey was composed by 28 questions concerning surgical team characteristics, technical preferences, surgical volume, results and available institutional resources. This questionnaire was designed to be brief, enabling completion in less than 10 min. A total of four reminders were weekly sent to non-responders. No incentives were offered to the participants that completed the questionnaire.

Laparoscopic resection included totally laparoscopic, hand-assisted and videoassisted procedures. Major hepatectomies were defined as resection of three or more segments. High-volume centers were defined as those with more than 50 hepatic resections per year.

All studied variables, except one, were categorical and were presented as frequency (percentage). Continuous variable was presented as the mean±standard deviation and median (range).

## RESULTS

The questionnaire was sent to 84 Brazilian liver surgeons from different centers. Of these, 43 study participants (51.2%) responded the survey (42 complete and one incomplete responses). Table 1 summarizes questions and answers in the survey.

**TABLE 1:** Questions applied in the Brazilian Liver Surgery Survey

1. Where is your center located? (Figure 1)	
2. Your institution offers:	
- State funded health care:	9 (20.9%)
- Private health care	10 (23.3%)
- State funded and private health care:	24 (55.8%)
3. How many surgeons perform liver resections in your team?	
- Mean: 3.37 (minimum: 1/ maximum: 6/ standard deviation: 1.33)	
4. Does your team have residency/fellowship programs? (Figure 2A)	
- Yes:	37 (86%)
- No:	6 (14%)
5. Does your team also perform liver transplantation?	
- Yes:	21 (48.8%)
- No:	22 (51.2%)
6. How many liver resections are performed per year in your center? (Figure 2B)	
- >15:	6 (14%)
- 16-30:	15 (34.8%)
- 31-50:	15 (34.8%)
- 51-80:	6 (14%)
- 81-100:	0 (0%)
- >100:	1 (2.3%)
7. What is the percentage of laparoscopic liver resections related to overall procedures? (Figure 2C)	
- 0%:	4 (9.3%)
- 1-9%:	17 (39.5%)
- 10-19%:	10 (23.3%)
- 20-39%:	6 (14%)
- 40-49%:	5 (11.6%)
- >50%:	1 (2.3%)
8. What is the main indication for liver resection in your institution?	
- Malignant primary tumours:	6 (14.3%)
- Benign tumours:	1 (2.4%)
- Liver metastases:	35 (83.3%)
9. What is the percentage of major hepatectomy?	
- 1-10%:	0 (0%)
- 11-20%:	5 (11.9%)
- 21-40%:	15 (35.7%)
- 41-50%:	17 (40.5%)
- >50%:	5 (11.9%)
10. What is the preferred incision for major hepatectomy?	
- Midline incision:	0 (0%)
- “J” incision:	27 (64.3)
- “Mercedes” incision:	5 (11.9%)
- Transverse incision:	0 (0%)
- Bilateral subcostal incision:	10 (23.8%)
- Long midline incision with transverse extension:	0 (0%)
- Other incisions:	0 (0%)
11. Do you use Pringle maneuver:	
- Routinely:	2 (4.8%)
- Selective:	37 (88.1%)
- Never:	3 (7.1%)
12. Do you use Pringle maneuver in major hepatectomy:	
- Routinely:	11 (26.2%)
- Selective:	29 (69%)
- Never:	2 (4.8%)
13. How is your preferred approach to hepatic pedicle?	
- Dissection of all structures:	17 (40.5%)
- Extrahepatic Glissonian approach:	9 (21.4%)
- Intrahepatic Glissonian approach:	7 (16.7%)
- Pedicle clamping (Pringle/ Hemi-Pringle) plus approach during parenchyma transection:	8 (19%)
- Other:	1 (2.4%)
14. What is your preferred method for parenchyma transection in OPEN surgeries?	
- Ultrasonic surgical aspirator:	14 (33.3%)
- Kelly clamp crushing:	10 (23.8%)
- Energy disposable devices (p.e. harmonic scalpel or advanced bipolar vessel sealing):	8 (19%)
- Water-jet dissection:	1 (2.4%)
- Bipolar cautery:	6 (14.3%)
- Silkclasy:	2 (4.8%)
- Digitoclasy:	0 (0%)
- Stapler:	0 (0%)
- Other:	1 (2.4%)
15. What is your preferred method for parenchyma transection in LAPAROSCOPIC surgeries?	
- Ultrasonic surgical aspirator:	4 (9.5%)
- Stapler:	3 (7.1%)
- Harmonic scalpel:	17 (40.5%)
- Advanced bipolar energy:	13 (31%)
- Other:	1 (2.4%)
- We do not perform laparoscopic resections:	4 (9.5%)
16. Do you have intraoperative ultrasound available?	
- Yes, for open procedures:	32 (76.2%)
- Yes, for open and laparoscopic procedures:	7 (16.7%)
- No:	3 (7.1%)
17. Do you use routinely drain in major hepatectomies?	
- Yes:	38 (90.5%)
- No:	4 (9.5%)
18. If you routinely use drain in major hepatectomies, what is your preferred drain?	
- Closed tubular in aspiration (i.e. Blake, Jackson-Pratt):	33 (78.6%)
- Open tubular and laminar (i.e. Waterman):	2 (4.8%)
- Open laminar (i.e. Penrose):	4 (9.5%)
- Open tubular:	3 (7.1%)
19. Do you apply routinely haemostatics or sealants on the raw surface area after hepatectomy?	
- No:	27 (64.3%)
- Yes, fibrin glue:	7 (16.7%)
- Yes, topic haemostatic (sponges, plaque, mesh):	8 (19%)
20. Do you perform intraoperative test for biliary leakage after major hepatectomies?	
- Always:	13 (31%)
- In selected cases:	19 (45.2%)
- Never:	10 (23.8%)
21. What is your incidence of postoperative biliary leakage?	
- <10%:	33 (78.6%)
- 10-19%:	9 (21.4%)
- 20-29%:	0 (0%)
- >30%	0 (0%)
22. What is you intraoperative blood transfusion rate?	
- < 10%:	23 (54.8%)
- 10-19%:	11 (26.2%)
- 20-29%:	3 (7.1%)
- 30-39%:	4 (9.5%)
- 40-49%:	0 (0%)
- >50%:	1 (2.4%)
23. What is your postoperative morbidity rate?	
- <10%:	10 (23.8%)
- 10-19%:	18 (42.8%)
- 20-29%:	7 (16.7%)
- 30-39%:	5 (11.9%)
- 40-49%:	2 (4.8%)
- >50%:	0 (0%)
24. What is your postoperative mortality rate? (Figure 2D)	
- <1%:	5 (11.9%)
- 1-3%:	23 (54.8%)
- 4-6%:	9 (21.4%)
- 7-10%:	5 (11.9%)
- >10%:	0 (0%)
25. Do you have percutaneous biliary drainage available in your hospital?	
- Yes:	38 (90.5%)
- No:	4 (9.5%)
26. Do you have portal embolization available in your hospital?	
- Yes:	37 (88.1%)
- No:	5 (11.9%)
27. Do you have transarterial chemoembolization available in your hospital?	
- Yes:	33 (78.6%)
- No:	9 (21.4%)
28. Do you have endoscopic retrograde cholangiopancreatography available in your hospital?	
- Yes:	41 (97.6%)
- No:	1 (2.4%)

**FIGURE 1:**
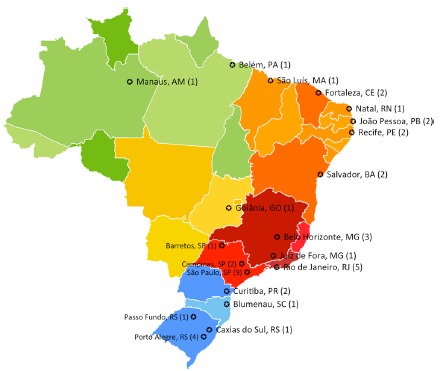
Map with the participant centers

Note: Number of centers in each city is between brackets

All five Brazilian regions are represented (Figure 1), as follows: South - nine answers (20.9%); Southeast - 21 answers (48.8%); Midwest - one answer (2.3%); North - three answers (7%); and Northeast - nine answers (20.9%). The majority of the answering centers are located in state capitals (86%). Twenty-four centers (55.8%) are public and private institutions; nine are exclusively public (20.9%), and ten (23.3%) private.

**FIGURE 2:**
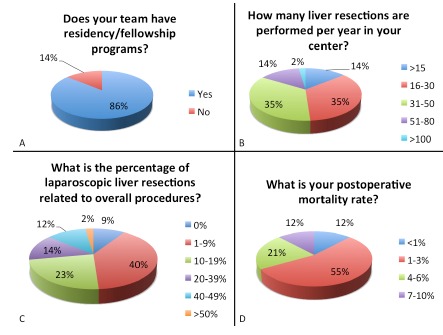
Most representative questions

The mean number of surgeons who perform liver resections in each team is 3.37 (1-6) and the majority (86%) has residents and/or fellows training in their groups (Figure 2A). Almost half of the answering groups (48.8%) are also involved in liver transplantation. Seven centers (16.3%) report a surgical volume larger than 50 liver resections per year, and only one team (2.3%) report more than 100 hepatectomies annually (Figure 2B).

More than 90% of liver surgery groups perform laparoscopic resections (Figure 2C); however, procedures by laparoscopy are still responsible for a little amount of surgeries (1-9% of laparoscopic procedures in 39.5% of groups). The main indication for liver resection is liver metastases (83.3%). Major hepatectomies are responsible for 20-49% of liver resections in most groups (66.2%) and the preferred incision in these cases is “J” shaped incision (64.2%), followed by bilateral subcostal incision (23.8%) and “Mercedes” incision (12%).

Pringle maneuver is used selectively by most surgeons (88.1%); however, in major hepatectomies, the selective use drops down to 69%, and the routine clamping application increases from 4.7-26.2%. The preferred approach to hepatic pedicle is done by dissection of all hilar structures (40.5%), followed by extrahepatic Glissonian approach in 21.4% of cases, pedicle clamping (Pringle/Hemi-Pringle) plus intrahepatic approach during parenchyma transection (19%) and intrahepatic Glissonian approach (16.7%)^9^.

In open surgeries, the chosen method for parenchyma transection is ultrasonic surgical aspirator (33.3%), followed by Kelly clamp crushing technique (23.8%), energy disposable devices (19%) and bipolar cautery (14.3%). For laparoscopic parenchyma transection, the preferred method is the harmonic scalpel (40.5%), followed by disposable devices using advanced bipolar energy (31%).

Most centers have only conventional intraoperative ultrasound probes (76.2%), while only 16.7% have access to both laparoscopic and open devices. Three groups (7.1%) do not have intraoperative ultrasound available.

Most surgeons do not use haemostatic or sealants on the raw surface after hepatectomy (64.3%). For those who use, the preferred is haemostatic patch (19%), followed by fibrin glues (16.7%).

For major hepatectomies, most surgeons routinely employ drainage (90.5%) being the preferred a tubular closed suction drain (78.6%), like Blake^®)^ or Jackson-Pratt^®)^. The use of intraoperative test for biliary leakage in major resections is done routinely by 31% of surgeons, while 45.2% applied in selected cases and 23.8% never do it.

Regarding outcomes, most surgeons report an incidence of biliary leakage <10% (78.6%), an intraoperative blood transfusion rate <10% (54.8%), a morbidity rate between 0-19% (66.7%) and a mortality rate (Figure 2D) ranging from 1-3% (54.8%).

In the evaluation of institutional resources, percutaneous biliary drainage, portal embolization, trans-arterial chemoembolization and endoscopic retrograde cholangiopancreatography are available for, respectively, 90.5%, 88.1%, 78.6% and 97.6% of groups.

## DISCUSSION

This is the first national survey about liver surgery in a Latin American country, until our best knowledge. It provides a comprehensive insight into the practice of liver surgery in Brazil. We had a reasonable response rate (51.2%) and all country regions were represented, showing that hepatic surgery is diffused around the country. Since liver surgery is a complex procedure, requiring a complex hospital structure, most of the centers are located at state capitals (86%). This can also be a reflex of a regionalization observed recently in other countries, such as United States^13^, with better results in high-volume hospitals. Only 23.3% of groups are exclusively associated to private healthcare, with 76.7% of groups offering state funded public healthcare, showing that hepatic surgery is largely available to the population in most of these cities.

The groups are small, but they are involved with residency or fellowship training programs, which represents an academic involvement in teaching and disseminating knowledge. It is important to emphasize the need of further training after a general surgery program to achieve excellence in HPB surgery; however, in South America, there are only 14 HPB fellowship programs registered in IHPBA^10^ mostly in Argentina and Brazil. Teaching hepatobiliary surgery is still a challenge in South American countries.

There is a worldwide trend to concentrate complex liver surgery in high-volume centers, because it is widely accepted that morbidity and mortality for major surgery correlates with the case-load of the hospital and the experience of the team^6^ ^,^ ^11^ ^,^ ^13^ ^,^ ^27^. However the exact number to consider a center as a high-volume institution is controversial, ranging from 10-110 hepatic procedures each year^6^ ^,^ ^7^ ^,^ ^11^ ^,^ ^13^ ^,^ ^27^. The International Hepato-Pancreato-Biliary Association (IHPBA) recommends at least 25 hepatic operations in the HPB fellowship^19^. Since there is no consensus in this matter, we decided for a cut-off of 50 procedures/year because it represents a mean of one liver resection/week and is close to American cut-offs for quality, which is 45 liver resections per year^7^ ^,^ ^13^. Pancreaticoduodenectomy, another complex abdominal procedure, has also a cut-off of 50 procedures/year for high-volume hospitals^14^.

In the last two decades minimally invasive liver surgery has been increasingly accepted. Its initial development was slow, held by 1) technical barriers, since translation of conventional techniques to laparoscopic approach was needed; 2) fear of anticipated intraoperative hazards, such as massive bleeding and the risk of gas embolism secondary to pneumoperitoneum; and 3) doubts on oncological outcomes such as adequate margins, port site seeding and long-term survival^8^. Our findings show that laparoscopic approach is disseminated around the country, being applied by more than 90% of groups, rates comparable to a worldwide survey that found 88% of centers employ laparoscopic approach in liver surgery^26^. However, this approach is responsible for a small portion of liver resections in most of the groups maybe due to the high cost of the equipment for minimally invasive surgery (laparoscopic ultrasound devices are available in only 16.7% of the centers). This is a clear barrier to development of laparoscopic surgery in Brazil.

Laparoscopic approach for liver resection is gaining space worldwide, especially in the treatment of benign tumors as hepatocellular adenoma^8^ ^,^ ^17^, which is more common in young women, and in the treatment of hepatocellular carcinoma^8^ ^,^ ^18^, with a likely advantage over conventional open approach. According to Kawaguchi et al.^21^, the relative number of 30% of minimally invasive approach for all liver resections is the average for specialized centers. An effort in training has to be made to achieve this numbers in the near future.

An interesting finding is that 90.5% of groups still use abdominal drains after major hepatectomies, although this contrasts with evidence from the Cochrane systematic review^15^, and the growing tendency toward fast-track and Enhanced Recovery After Surgery (ERAS) programs^31^, which substantially disagreed with the routine use of surgical drains. Despite evidence on this matter, a recent Italian survey in liver surgery also revealed 93% of drainage following hepatic resection^1^.

Intraoperative blood loss is a significant factor affecting the short- as well as long-term outcomes after liver resection and efforts to avoid it should be done^9^ ^,^ ^25^. The most employed technique to control liver inflow is hepatic pedicle clamping, called Pringle maneuver^28^. In an Italian liver surgery survey, it was performed in 56.4% of centers^1^. Other multi-institutional survey found that most centers applied Pringle’s maneuver routinely (50%) or when excessive bleeding occurs (43%) during open hepatectomy^26^. Our study showed a selective use of Pringle maneuver by Brazilian surgeons (88.1%) in overall liver resections, with increased use of routine pedicle clamping in major resections (26.2%).

Hepatic pedicle control is a key point for liver resection. There is a worldwide tendency to dissect the hepatic artery and the portal vein individually, according to data published by Mise et al.^26^ (48% for dissection vs. extrahepatic Glissonian approach in 33%); our survey showed a similar pattern (40.5% hilar structures dissection vs. 21.4% extrahepatic Glissonian approach). The advantage of the en-bloc extrafascial pedicle approach is that the liver can be separated into three sections by simply clamping the secondary Glissonian pedicle after an extrahepatic approach without prolonged liver dissection at the hepatic hilum^32^.

Despite introduction of many devices to transect liver parenchyma in the last years, a conservative trend was observed in our study. Clamp-crushing technique and ultrasonic dissectors (CUSA), which were introduced, respectively, in the 1970s^24^ and in the 1980s^30^, were the two favorite methods by 23.8% and 33.3% of surgeons, respectively. The Brazilian experience is a reflex of a worldwide inclination, where these two methods are also preferable^26^. Worlds´ preference reinforces the evidence from randomized trials showing absence of superiority regarding new devices over the classical methods^23^.

Our study has some limitations. The first is a moderate response rate (51.2%), hampering access to a more complete view of the national scenario. This rate is below other world liver surgery surveys, which reached 75% of responders^6^ ^,^ ^20^. It is the nature of any survey that only part of the population is approached and only part of the surveyed surgeons may reply^6^. Other limitation is a potential selection bias, since most of the responders were from Brazil southeast region (48.8%), perhaps reflecting a regional pattern.

## CONCLUSION

Despite its limitations, this is the first depiction of liver surgery in Brazil. All authors are continuously involved in academic and societary interaction through CB-IHPBA. Thus, we do know much about regional practices in our country and, importantly, the results left the authors with a pleasant feeling of a faithful portrait. The study covered all regions of the country through its responders and indicates a surgical practice aligned with worldwide excellence centers, concentrated on hospitals dedicated to academic practice. This is probably the best conclusion our paper offers: liver surgery in Brazil has taken the right path.

## References

[1] Aldrighetti L, Belli G, Boni L, Cillo U, Ettorre G, De Carlis L (2015). Italian experience in minimally invasive liver surgery a national survey. Updates Surg.

[2] Amico EC, Alves JR, João SA, Guimarães PL, Medeiros JA, Barreto ÉJ (2016). Immediate complications after 88 hepatectomies - brazilian consecutive series. Arq Bras Cir Dig.

[3] Araujo RL, Cesconetto D, Jeismann VB, Fonseca GM, Coelho FF, Kruger JA (2016). Central hepatectomy for biliary cystadenoma parenchyma-sparing approach for benign lesions. Arq Bras Cir Dig.

[4] Asiyanbola B, Chang D, Gleisner AL, Nathan H, Choti MA, Schulick RD (2008). Operative mortality after hepatic resection are literature-based rates broadly applicable?. J Gastrointest Surg.

[5] Bismuth H, Eshkenazy R, Arish A (2011). Milestones in the evolution of hepatic surgery. Rambam Maimonides Med J.

[6] Breitenstein S, Apestegui C, Petrowsky H, Clavien PA (2009). "State of the art" in liver resection and living donor liver transplantation: a worldwide survey of 100 liver centers. World J Surg.

[7] Buettner S, Gani F, Amini N, Spolverato G, Kim Y, Kilic A (2016). The relative effect of hospital and surgeon volume on failure to rescue among patients undergoing liver resection for cancer. Surgery.

[8] Coelho FF, Kruger JA, Fonseca GM, Araujo RL, Jeismann VB, Perini MV (2016). Laparoscopic liver resection Experience based guidelines. World J Gastrointest Surg.

[9] de Boer MT, Molenaar IQ, Porte RJ (2007). Impact of blood loss on outcome after liver resection. Dig Surg.

[10] de Santibanes M, de Santibanes E, Pekolj J (2016). Training in hepato-pancreato-biliary surgery during residency past, present and future perspectives. J Hepatobiliary Pancreat Sci.

[11] Dimick JB, Wainess RM, Cowan JA, Upchurch GR, Knol JA, Colletti LM (2004). National trends in the use and outcomes of hepatic resection. J Am Coll Surg.

[12] Fernandes ES, Mello FT, Ribeiro-Filho J, Monte-Filho AP, Fernandes MM, Coelho RJ (2016). The largest western experience with hepatopancreatoduodenectomy lessons learned with 35 cases. Arq Bras Cir Dig.

[13] Gani F, Azoulay D, Pawlik TM (2017). Evaluating Trends in the Volume-Outcomes Relationship Following Liver Surgery Does Regionalization Benefit All Patients the Same?. J Gastrointest Surg.

[14] Gordon TA, Bowman HM, Tielsch JM, Bass EB, Burleyson GP, Cameron JL (1998). Statewide regionalization of pancreaticoduodenectomy and its effect on in-hospital mortality. Ann Surg.

[15] Gurusamy KS, Samraj K, Davidson BR (2007). Routine abdominal drainage for uncomplicated liver resection. Cochrane Database Syst Rev.

[16] Harris PA, Taylor R, Thielke R, Payne J, Gonzalez N, Conde JG (2009). Research electronic data capture (REDCap)--a metadata-driven methodology and workflow process for providing translational research informatics support. J Biomed Inform.

[17] Herman P, Coelho FF, Perini MV, Lupinacci RM, D'Albuquerque LA, Cecconello I (2012). Hepatocellular adenoma an excellent indication for laparoscopic liver resection. HPB (Oxford).

[18] Herman P, Lopes Fde L, Kruger JA, Fonseca GM, Jeismann VB, Coelho FF (2016). IS RESECTION OF HEPATOCELLULAR CARCINOMA IN THE ERA OF LIVER TRANSPLANTATION WORTHWILE A single center experience. Arq Gastroenterol.

[19] IHPBA (2017). Standards for Hepato-Pancreato-Biliary Training [Internet].

[20] Jrearz R, Govindarajan A, Jayaraman S (2017). A survey of current practices and barriers to expanding laparoscopic HPB surgery in Canada. HPB (Oxford).

[21] Kawaguchi Y, Hasegawa K, Wakabayashi G, Cherqui D, Geller DA, Buell JF (2016). Survey results on daily practice in open and laparoscopic liver resections from 27 centers participating in the second International Consensus Conference. J Hepatobiliary Pancreat Sci.

[22] Kelley K, Clark B, Brown V, Sitzia J (2003). Good practice in the conduct and reporting of survey research. Int J Qual Health Care.

[23] Lesurtel M, Selzner M, Petrowsky H, McCormack L, Clavien PA (2005). How should transection of the liver be performed?: a prospective randomized study in 100 consecutive patients: comparing four different transection strategies. Ann Surg.

[24] Lin TY (1974). A simplified technique for hepatic resection the crush method. Ann Surg.

[25] Margonis GA, Kim Y, Samaha M, Buettner S, Sasaki K, Gani F (2016). Blood loss and outcomes after resection of colorectal liver metastases. J Surg Res.

[26] Mise Y, Sakamoto Y, Ishizawa T, Kaneko J, Aoki T, Hasegawa K (2013). A worldwide survey of the current daily practice in liver surgery. Liver Cancer.

[27] Nathan H, Cameron JL, Choti MA, Schulick RD, Pawlik TM (2009). The volume-outcomes effect in hepato-pancreato-biliary surgery hospital versus surgeon contributions and specificity of the relationship. J Am Coll Surg.

[28] Pringle JH (1908). V Notes on the Arrest of Hepatic Hemorrhage Due to Trauma. Ann Surg.

[29] Surjan RCT, Makdissi FF, Machado MAC (2015). Anatomical basis for the intrahepatic glissonian approach during hepatectomies. Arq Bras Cir Dig.

[30] Tranberg KG, Rigotti P, Brackett KA, Bjornson HS, Fischer JE, Joffe SN (1986). Liver resection A comparison using the Nd-YAG laser, an ultrasonic surgical aspirator, or blunt dissection. Am J Surg.

[31] Wong-Lun-Hing EM, van Dam RM, van Breukelen GJ, Tanis PJ, Ratti F, van Hillegersberg R (2017). Randomized clinical trial of open versus laparoscopic left lateral hepatic sectionectomy within an enhanced recovery after surgery programme (ORANGE II study). Br J Surg.

[32] Yamamoto M, Katagiri S, Ariizumi S, Kotera Y, Takahashi Y (2012). Glissonean pedicle transection method for liver surgery (with video). J Hepatobiliary Pancreat Sci.

